# Synthesis of Polymers From Bio‐Based Methacrylates Comprising Aromatic or Aliphatic Structures Using NIR‐Mediated ATRP

**DOI:** 10.1002/marc.202500718

**Published:** 2025-11-09

**Authors:** Nicolai Meckbach, Lea Viktoria Rubbert, Bernd Strehmel, Veronika Strehmel

**Affiliations:** ^1^ Institute for Coatings and Surface Chemistry Faculty of Chemistry Niederrhein University of Applied Sciences Krefeld Germany

**Keywords:** bio‐based methacrylates, block copolymers, cyanines, homopolymers, NIR‐mediated ATRP

## Abstract

NIR‐mediated ATRP is used for the synthesis of polymethacrylates from the bio‐based monomers 4‐(4‐methacryloyloxyphenyl)butan‐2‐one and methyl 9‐hydroxy‐10‐(methacryloyloxy) octadecanoate / methyl 9‐(methacryloyloxy)‐10‐hydroxy octadecanoate. According to the NIR‐mediated ATRP protocol, excitation is carried out at 790 nm in the presence of copper(II)bromide tris(2‐pyridylmethyl)amine complex as deactivator and a heptamethine cyanine comprising a barbiturate group in the meso‐position operating as sensitizer. The homopolymers obtained are compared with similarly made poly(methyl methacrylate) regarding yield, molecular weight, dispersity, and glass transition temperature. Furthermore, block copolymers made from methyl methacrylate and the bio‐based monomers are included in this comparison. Dispersity is broader in the case of the bio‐based polymethacrylates compared to poly(methyl methacrylate), indicating a higher contribution of chain termination during NIR‐mediated ATRP using the bio‐based methacrylates. Notably, all macroinitiators are chain extendable. In addition, the block copolymers synthesized from the bio‐based methacrylates significantly differ regarding their solubility, that is caused by the distinct structural features in the ester part of these monomers. Results obtained from GPC investigation of tetrahydrofuran soluble copolymers are included in the discussion. These results show that the initiation efficiency is lower for the methyl 9‐hydroxy‐10‐(methacryloyloxy) octadecanoate / methyl 9‐(methacryloyloxy)‐10‐hydroxy octadecanoate isomer mixture compared to methyl methacrylate.

## Graphical abstract

Two bio‐based methacrylates (4‐(4‐methacryloyloxyphenyl)butan‐2‐one and methyl 9‐hydroxy‐10‐(methacryloyloxy) octadecanoate / methyl 9‐(methacryloyloxy)‐10‐hydroxy octadecanoate isomer mixture) are investigated in Photo‐ATRP to obtain homopolymers and block copolymers. The commercially available methyl methacrylate is selected for comparison of homopolymer and copolymer synthesis regarding yield, molecular weight and dispersity in case of tetrahydrofurane soluble polymers, and glass transition temperature.

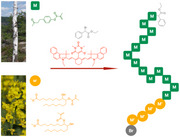

## 1 Introduction

Reversible deactivation radical polymerization (RDRP) has received increased importance in polymer synthesis because it benefits to tailor the size of the polymer and opens the possibility to make block copolymers [1, 2, 3, 4]. According to a IUPAC report, this polymerization method belongs to the emerging applications having the potential to make our planet more sustainable [5]. Atom Transfer Radical Polymerization (ATRP) [2, 6, 7, 8, 9], nitroxide‐mediated radical polymerization (NMP) [10, 11], and reversible addition fragmentation transfer polymerization (RAFT) [12, 13, 14, 15] follow different reaction mechanisms to control the molecular weight and dispersity of the polymers. Further research on these controlled radical polymerizations has been carried out to extend the controlled radical polymerization scheme to a broader variety of monomers on the one hand and to receive a narrow molecular weight distribution of both the homopolymers and block copolymers obtained on the other hand. Among the different RDRP reaction schemes, ATRP has developed as a versatile method to synthesize polymers with tailored size. First contributions reported the use of a much higher concentration of the copper catalyst CuX/**L** (X: Cl^−^, Br^−^; **L**: amine ligand) operating there as the activator in the traditional ATRP [6, 16]. Consequently, research focused on the reduction of copper content, resulting in various controlled radical polymerization protocols, including, for example, activators regenerated by electron transfer (ARGET) ATRP [17, 18], supplemental activator and reducing agent (SARA) ATRP [19], electrochemically mediated ATRP (eATRP) [20, 21], mechanically induced ATRP (mechano‐ATRP) [22, 23], ultrasonication‐induced ATRP (sono‐ATRP) [24], and photo‐induced ATRP (photo‐ATRP) [22, 25, 26, 27, 28, 29, 30, 31, 32, 33, 34, 35]. Here, the deactivator CuX_2_/**L** served as substrate to generate the activator, CuX/**L,** requesting a much lower amount of deactivator.

Light‐driven ATRP systems, named as photo‐ATRP, result in a significantly lower amount of deactivator than that used in the classical ATRP protocol [3, 16, 25, 36, 37, 38, 39, 40, 41]. Recent work reported the use of less than 10 ppm deactivator in photo‐ATRP setups [1, 42]. The light‐driven ATRP protocols can be pursued by direct excitation of the deactivator [38] or by a sensitizer (**Sens**) enabling for excitation the entire visible light spectrum and, of course, also near‐infrared (NIR) radiation with wavelengths up to 850 nm in case of NIR‐mediated ATRP [29, 34, 35, 43, 44]. Metal‐free ATRP protocols have additionally received attention since these reactions result in polymers comprising no traces of copper ions [25, 26, 30, 45, 46, 47, 48, 49, 50]. This can be beneficial for several applications. However, this methodology was not considered with the monomers used in this study because NIR mediated ATRP requires operating with CuX_2_/**L** as cocatalyst [29].

Moreover, bio‐based monomers have received increasing attention regarding their suitability not only as substitutes for traditional monomers but also to meet the requirements of future materials, including improved tailor‐made properties of the polymers, environmental friendliness during polymer manufacturing, application, and at the end of the polymer lifetime, which means reusability and recyclability. Various unsaturated fatty acids and phenolic compounds represent examples for starting materials for monomer synthesis that can be obtained from plant materials, which are considered as renewable resources [48, 51, 52, 53, 54, 55, 56, 57, 58, 59, 60, 61, 62, 63]. Bio‐based monomers have already moved into the focus of investigation by a metal‐free ATRP approach [48]. The combination of bio‐based monomers and light as a manufacturing tool in the photoinitiated polymerization operating on demand makes polymerization of bio‐based monomers more efficient from a green chemistry point of view [9].

This work discusses the behavior of two bio‐based methacrylates in a NIR‐mediated ATRP setup in comparison with the widely used commercial methyl methacrylate. The longer alkyl chain containing in the aliphatic bio‐based methacrylate might have an influence on the activation and deactivation rate constants of the ATRP scheme that may differ from both the commercial methyl methacrylate and the bio‐based methacrylate comprising an aromatic structure. NIR radiation excludes any inner filter effects of the materials used for the polymerization. Furthermore, the molecular weight and dispersity of the homopolymers and tetrahydrofuran soluble copolymers, as well as the glass transition temperature of both homopolymers and selected copolymers, are included in the discussion as well.

## 2 Results and Discussion

### 2.1 Selection of Materials and Mechanistic Aspects

**FIGURE 1:**
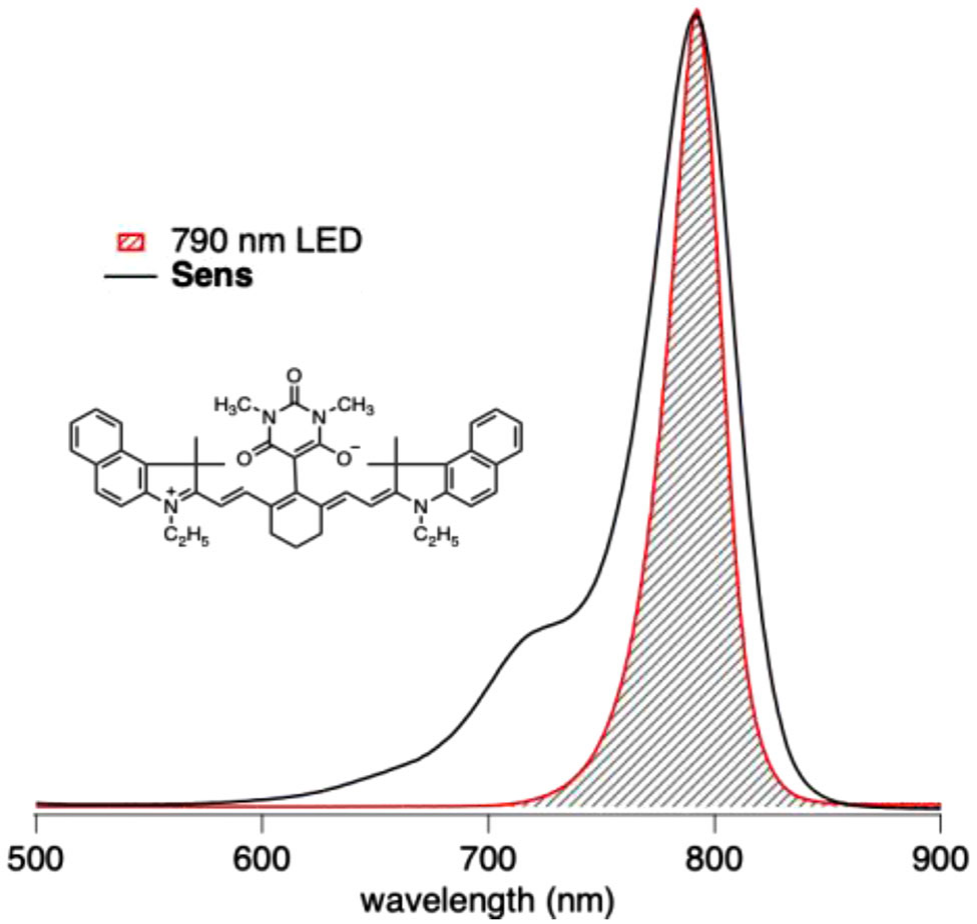
Chemical structure of the sensitizer (**Sens**) and absorption profile of **Sens** in comparison with the emission profile of the LED (790 nm) used. The hatched area shows the spectral overlap between the absorption of **Sens** and the emission profile of the LED.

The first report connected to NIR‐sensitized controlled photopolymerization using a heptamethine cyanine as sensitizer and a 790 nm LED for irradiation appeared in 2018 [29]. This study approved the functional operation of the sensitizer (**Sens**) comprising a barbiturate group in the *meso*‐position. Replacement by thiobarbiturate failed. Thus, the structure shown for **Sens** in Figure 1 operated in this study. Preliminary studies showed good compatibility of **Sens** in organic surroundings with low tendency of H‐aggregate formation [35]. This sensitizer shows good overlap between its UV–vis‐NIR absorption spectrum with the emission profile of the NIR LED (λ_em_ = 790 nm) used (Figure 1). Therefore, this system can be seen as state‐of‐the‐art for NIR‐mediated ATRP with radiation around 800 nm, considering the availability of the sensitizer at a larger scale.

For the sake of simplicity, the individual reactions were split into a part involving **Sens** [1, 2, 3, 4, 5, 6, 7], Equations (1), (2), (3), (4), (5), (6), (7), and those disclosing the general ATRP protocol [8, 9, 10, 11]. Excitation of **Sens** with light of the LED results in the formation of its excited singlet state Sens*, Equation 1. There does not exist any report regarding the formation of a triplet state of **Sens**. Its first excited singlet state Sens* possesses several reaction possibilities. This can be a competing photoinduced electron transfer (**PET**) between Sens* with either an alkyl bromide P_n_‐Br (n≧1), Equation 2, or CuBr_2_/L (**L** = amine ligand), Equation 3, resulting in formation of the activator CuBr/L for the ATRP. Equation 2 would be the initial step of a metal‐free ATRP based on an oxidative protocol. This oxidative protocol did not effectively proceed with the NIR sensitizer **Sens** depicted in Figure 1 which follows a controlled polymerization protocol [29, 43]. The isolated polymer indicated a rather conventional radical polymerization mechanism. IUPAC depreciates further use of free radical polymerization for this polymerization mechanism [64].

(1) $$\mathrm{Sens}\;\overset{h\nu }{\to }\;\mathrm{Sen}s^{*}$$

(2) $$\mathrm{Sen}s^{*}+P_{n}-\mathrm{Br}\;\overset{k_{r1}}{\to }\;\mathrm{Sen}s^{+•}+P_{n}\cdot +Br^{-}$$

(3) $$\mathrm{Sen}s^{*}+\mathrm{CuB}r_{2}/L\;\overset{k_{r2a}}{\to }\;\mathrm{Sen}s^{+•}+\mathrm{CuBr}/L+Br^{-}$$

(4) $$\mathrm{Sens}+\mathrm{CuB}r_{2}/L\;⇆\;[\mathrm{Sens}\text{…}\mathrm{CuB}r_{2}/L]$$

(5) $$K=\frac{[\mathrm{Sens}\text{…}\mathrm{CuB}r_{2}/L]}{\left[\mathrm{Sens}\right]\left[\mathrm{CuB}r_{2}/L\right]}\;$$

(6) $$[\mathrm{Sens}\text{…}\mathrm{CuB}r_{2}/L]\;\overset{h\nu }{\to }\;[\mathrm{Sens}\text{…}\mathrm{CuB}r_{2}/L]*$$

(7) $$[\mathrm{Sens}\text{…}\mathrm{CuB}r_{2}/L]*\;\overset{k_{r2b}}{\to }\;\mathrm{Sen}s^{+•}+\mathrm{CuBr}/L+Br^{-}$$

UV–vis‐NIR spectroscopic titration shows formation of a 1:1 complex based on the equilibrium depicted in Equation 4, indicating formation of a new hypsochromic shifted absorption band exhibiting a maximum at 479 nm. The complex formed possesses a broad absorption, which covers the emission profile of the NIR‐LED emitting at 790 nm that is shown in Figure 1. Thus, one needs to consider both the single molecule **Sens** and its complex (**Sens∙∙∙CuBr_2_/L**) formed in the equilibrium with the deactivator CuBr_2_/L as expressed by the equilibrium constant defined in Equation 5. Both **Sens** (Equation 1) and **Sens∙∙∙CuBr_2_/L** (Equation 6) can contribute to the entire reaction protocol. The excited state of the complex, (**Sens∙∙∙CuBr_2_/L**)*, can also form the activator, Equation 7.

**FIGURE 2:**
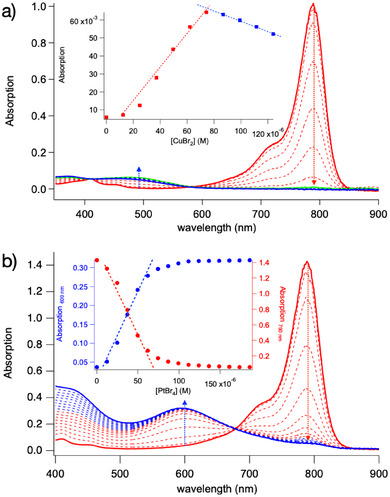
UV/VIS/NIR Titration of **Sens**. a) Using increasing concentration of CuBr_2_ in DMF that showed a hypsochromic shift (↓790 nm, ↑500 nm) and an isosbestic point at ∼580 nm, indicating 1:1 complex formation as described in Equation 4. Inset a) Absorption at 790 nm as a function of CuBr_2_ concentration. b) Using PtBr_4_ in DMF that showed a new hypsochromic shifted band at 600 nm and an isosbestic point at 679 nm, indicating 1:1 complex formation with Pt(IV). Inset b) Absorption at 790 nm (red) and 600 nm (blue) as a function of CuBr_2_ concentration.

Figure 2a,b illustrates changes in the absorption spectrum of **Sens** upon addition of CuBr_2_ (Figure 2a) and PtBr_4_ (Figure 2b) as alternative metal halide, respectively. Both exhibit a new hypsochromic shifted absorption band, which is in contrast to a previous report, where titration with FeBr_3_ indicated a bathochromic shifted absorption [34]. Nevertheless, all three metal ions show formation of a 1:1 complex with distinct absorption, indicating different electron density in the unsaturated moiety of **Sens** in the complex formed. This might influence the reaction rates *k*_r2a_ (Equation 3) and *k*_r2b_ (Equation 7). A high activator concentration results in a higher concentration of polymer radicals (P_n_∙), and thus, a higher contribution of chain termination.

The higher contribution of termination becomes clearer considering the individual steps of ATRP (Equations (8), (9), (10), (11)). Equation 8 discloses the activation of an alkyl bromide (P_n_‐Br; a macroinitiator made by n monomer units with n≧1) by the activator CuBr/L resulting in formation of polymer radical P_n_∙ and deactivator CuBr_2_/L. α‐Bromo(phenyl acetate) (**EBPA**) operates in the first step whose reaction constant with the activator is quite efficient to start the ATRP cycle [65]. The ligand **(L**), tris(2‐pyridylmethyl)amine (**TPMA**), complexes with CuBr_2_ to make it compatible with organic surroundings. It also adjusts the standard potential of the Cu(I)/Cu(II) equilibrium to achieve optimal conditions for controlled polymerization with optimally negligible contribution of termination. The polymer radicals formed propagate as shown in Equation 9 resulting in chain extended P_n+m_∙. The deactivator CuBr_2_/**L** possesses two possibilities to react either with P_n+m_∙ as described in Equation 10 and or with P_n+m_∙ and **Sens^+∙^** as shown in Equation 11 to yield back the activator CuBr/**L**. In the case that the reactions described in Equation 10 and Equation 11 dominate, one should expect a higher amount of activator compared to the case that only the reaction occurs, which is described in Equation 3 and always continuously delivers activator.

Furthermore, a higher concentration of polymer radicals can contribute to a higher amount on termination (Equation 12), resulting in higher molecular weight of the polymer. Moreover, there should also proceed a continuous formation of polymer in the dark period while **Sens** bleaches. On the other side, the reaction shown in Equation 11 demonstrates back formation of **Sens**. This can enter a new cycle in Equation 1 with no bleaching, as shown previously [29]. Therefore, no reaction should occur in the dark because all deactivator and **Sens** are formed back.

(8) $$\mathrm{CuBr}/L+P_{n}-\mathrm{Br}\overset{k_{a}}{\to }\;\mathrm{CuB}r_{2}/L+P_{n}^{•}$$

(9) $$P_{n}^{•}+mM\overset{k_{p}\;}{\to }\;\;P_{n+m}^{•}$$

(10) $$\;\mathrm{CuB}r_{2}/L+P_{n+m}^{•}\overset{k_{d1}}{\to }\;\mathrm{CuBr}/L+P_{n+m}-\mathrm{Br}$$

(11) $$\;\mathrm{CuB}r_{2}/L+P_{n+m}^{•}+\mathrm{Sen}s^{+•}\overset{k_{d2}}{\to }\;\mathrm{CuBr}/L+P_{n+m}-\mathrm{Br}+Sens$$

(12)

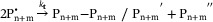




**FIGURE 3:**
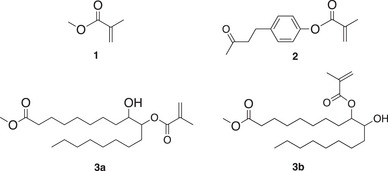
Chemical structure of the monomers methyl methacrylate (**1**), 4‐(4‐methacryloyloxyphenyl)butan‐2‐one (**2**), and methyl 9‐hydroxy‐10‐(methacryloyloxy) octadecanoate / methyl 9‐(methacryloyloxy)‐10‐hydroxy octadecanoate isomer mixture (**3a**/**3b**).

The bio‐based methacrylates 4‐(4‐methacryloyloxyphenyl)butan‐2‐one (**2**) and methyl 9‐hydroxy‐10‐(methacryloyloxy) octadecanoate / methyl 9‐(methacryloyloxy)‐10‐hydroxy octadecanoate isomer mixture (**3a**/**3b**) depicted in Figure 3 significantly differ in their structure from methyl methacrylate (**1**) that has been already investigated in photo‐ATRP [29]. Methyl methacrylate (**1**) also serves as a good reference to compare the polymerization behavior of the bio‐based monomers **2** and **3a**/**3b** resulting in new materials.

The bio‐based monomer **2**, derived from raspberry ketone, comprises the methacrylate structure directly connected to an aromatic ring substituted with an additional alkyl‐keto substituent at the *p*‐position. The crystalline monomer **2** shows a melting point at 29°C. In contrast to this, the bio‐based methyl 9‐hydroxy‐10‐(methacryloyloxy) octadecanoate / methyl 9‐(methacryloyloxy)‐10‐hydroxy octadecanoate isomer mixture (**3a**/**3b**) contains the methacrylate group as a substituent in the middle of a longer aliphatic chain and a hydroxy group at the alpha position to the methacrylate substituent. The isomer mixture **3a**/**3b** exhibits semicrystallinity with a glass transition temperature (*T*_g_) at −67°C, recrystallization above the glass transition, and melting of the crystal structures formed at −10°C. The aromatic moiety in **2** contributes to a higher solid liquid transition. The longer alkyl substituents in **3a**/**3b** may cause self‐organization, resulting in larger assemblies, which may influence the efficiency of the reactions described in Equations (9), (10), (11). The structural differences of the monomers **2** and **3a**/**3b** may affect the photo‐ATRP protocol. Therefore, the commercial monomer **1** was selected for comparison of photo‐ATRP investigation of the bio‐based methacrylates **2** and **3a**/**3b**.

### 2.2 Homopolymerization of the Bio‐Based Methacrylates in Comparison with Methyl Methacrylate Using Photo‐ATRP

The use of methyl methacrylate (**1**) in the NIR‐mediated ATRP resulted in poly(methyl methacrylate) (**poly‐1**) with a degree of polymerization (*X*_n_) of 125 and a dispersity (*Ð*) of 1.2 after an irradiation time of 24 h (Table 1), confirming previous findings [29]. Comparable polymerization conditions were selected for **2,** resulting in poly(4‐(4‐methacryloyloxyphenyl)butan‐2‐one) (**poly‐2**). The yield on homopolymer, degree of polymerization (*X*_n_), and dispersity (*Ð*) notably exhibit higher values for **poly‐2** compared to **poly‐1** after an irradiation time of 24 h (Table 1). Higher yield and higher degree of polymerization of **poly‐2** compared to **poly‐1** indicate a higher reactivity of the bio‐based aromatic monomer **2** compared to the commercial methyl methacrylate (**1**) in NIR‐mediated ATRP. Interestingly, the yield on **poly‐2** after 6 h polymerization time shows an approximately doubled value compared to the yield of **poly‐1** after 24 h polymerization time, indicating a significantly higher reactivity of the bio‐based aromatic monomer **2**. However, the degree of polymerization is lower, and dispersity is also higher for **poly‐2** after 6 h irradiation time compared to **poly‐1** after 24 h irradiation time. As a result, the structural moieties in **2** may connect to a higher fraction of termination as explained by the increase of *Ð*. Comparison of the NIR‐mediated ATRP of **2** at longer reaction time (24 h) with the polymerization at shorter polymerization time resulted in a moderate increase of yield, a notable increase of *X*_n_, and slightly higher dispersity of **poly‐2** at longer irradiation time (Table 1). Repetition of experiments shows comparable results concluded by the consideration of the **poly‐2** samples *batch a* and *batch b*. The initiation efficiency (*I*_eff_) (Equation 13) [6, 66] dropped down because the polymer radicals competitively terminate by either disproportionation or recombination (Equation 12), resulting in an increase of *Ð*.

(13) $$I_{\mathrm{eff}}=\frac{M_{n}^{\mathrm{th}}}{M_{n}}=\frac{\frac{{\left[\mathrm{monomer}\right]}_{0}}{{\left[\mathrm{initiator}\right]}_{0}}\;\times \;x\;\times \;M_{n}+M_{\mathrm{initiator}}}{M_{n}}$$

($M_{n}^{\mathrm{th}}$ = theoretical number‐average molecular weight, $M_{n}$ = experimentally determined number‐average molecular weight, *x* = conversion, [monomer]_0_ = monomer concentration at the beginning of the polymerization, [initiator]_0_ = initiator concentration at the beginning of the polymerization)

**TABLE 1:** Yield, theoretical number average molecular weight (*M*_n_^th^), experimentally determined number average molecular weight (*M*_n_), dispersity (*Ð*), degree of polymerization (*X*_n_), and glass transition temperature (*T*_g_) of polymethacrylates (**poly‐1**, **poly‐2**, and **poly‐3**) made by NIR‐mediated ATRP using different irradiation time (*t*), and initiation efficiency (*I*_eff_). **TPMA**: Tris(2‐pyridylmethyl)amine, **TBABr**: Tetra butyl ammonium bromide.

run	polymer	cocatalyst	t (h)	$M_{n}^{\mathrm{th}}$ (g/mol)	Yield (%)	M_n_ (g/mol)	*Ð*	*X*_n_	*I*_eff_ (g/mol)	*T*_g_ (°C)
1	**poly‐1**	**CuBr_2_**/**TPMA**	24	9543	31	12 551	1.2	125	0.76	122
2	**poly‐2**	**CuBr_2_**/**TPMA**	6	20 043	66	16 501	1.4	71	1.21	80
3	**poly‐2** (batch a)	**CuBr_2_**/**TPMA**	24	25 443	84	47 977	1.8	208	0.53	
4	**poly‐2** (batch b)	**CuBr_2_**/**TPMA**	24	12 393	81	42 083	1.6	182	0.29	
5	**poly‐3**	**CuBr_2_**/**TPMA**	48	11 643	38	90 088	1.4	226	0.13	−33
6	**poly‐1**	**PtBr_4_/TBABr** ^a^	3	9543	31	95 000	1.8	950	0.1	
7	**poly‐1**	**PtBr_4_/TBABr** ^b^	3	17 043	56	58 000	1.7	580	0.29	
8	**poly‐1**	**PtBr_4_/TBABr** ^c^	3	2043	6	21 400	1.5	214	0.1	

^a^ **TBABr** operated as ligand in a concentration of 75 µM and a loading of PtBr_4_ of 225 µM;

^b^ similar conditions as in run 6 but it operated with double amount on PtBr_4_;

^c^ similar conditions as in run 6 but under air.

The *I*_eff_ values document a certain dependence on conversion. An increase in viscosity can explain these findings. For example, monomer **2** appears to operate with higher initiation efficiency after a polymerization time of 6 h compared to a longer polymerization time (24 h) of both monomers **1** and **2** as concluded by consideration of Table 1 (run1, run2, and run3). A longer reaction time (24 h) led to a drop of *I*_eff_ for monomer **2** as shown in Table 1, run3 and run4. Comparison of the molecular weight of **poly‐2** obtained by photo‐ATRP (Table 1) with the results of the previous investigation of conventional radical polymerization of **2** showed a much higher molecular weight in the conventional radical polymerization [59]. The oxobutyl group in the *para* position at the phenyl ring may be responsibly cause the formation of much higher *M*_n_ that is concluded from results obtained for phenyl methacrylate that was polymerized under comparable conditions [59].

Furthermore, the bio‐based aliphatic monomer **3** needs a significant longer reaction time to achieve an acceptable yield of poly‐**3** (Table 1, run 5) compared to the methacrylates **1** and **2**. It assigns to less reactivity of **3** that may be caused by the long alkyl branches in the ester part of **3**. Steric reasons or self‐organization of the alkyl chains in **3** may explain the lower reactivity. Additional research is required to explore this hypothesis in more detail. However, this is not in the focus of this contribution and requires molecular modeling of the contributing structures. The lower initiation efficiency determined for **3** (Table 1, run 5) underlines a higher contribution of termination rather than controlled radical polymerization following the ATRP cycle as described in the Equations (1), (2), (3), (4), (5), (6), (7), (8), (9), (10), (11). Table 1 additionally shows the glass transitions temperatures of the homopolymers **poly‐1, poly‐2, and poly**‐**3**. In this series **poly‐3** exhibits the lowest *T*_g_ which increases of about 30 K with respect to the monomer **3**. Thus, the chains in this polymer appear quite flexible in contrast to **poly‐2** whose *T*_g_ appeared at 80°C. In case of **poly‐2**, the aromatic moieties mostly contribute to more stiffness connecting to a significant increase of *T*_g_. The reference polymer **poly‐1** still exhibits the highest *T*_g_.

Change to PtBr_4_ resulted in higher number average molecular weight (*M*_n_) and higher dispersity at a shorter polymerization time indicating a higher contribution of termination (Table 1, run 6). Doubling of the cocatalyst loading gave a decrease of *M*_n_ but a higher initiation efficiency concluded by comparison the data shown in Table 1, run 7 and run 6, respectively. The polymer obtained under air (Table 1, run 8) was chain extendable resulting in higher *M*_n_ of the chain extended **poly‐1** (*M*_n_ = 86.5 kg/mol) and a dispersity of 1.8 for the chain extended **poly‐1**. Interestingly, this cocatalyst also operated under air. Applying the exposure condition used in Table 1, run 6 but using aerobic conditions (Table 1, run 8) gave a lower molecular weight of the **poly‐1** (*M*_n_ = 21.4 kg/mol), and lower dispersity (1.5). This shows reduction of both molecular weight and dispersity in the presence of air (Table 1, run 8) compared to application of anaerobic conditions (Table 1, run 6). The polymer obtained under air (Table 1, run8) was also chain extendable under aerobic conditions resulting in higher molecular weight and higher dispersity (*M*_n_ = 30.9 kg/mol, *Ð* = 1.7). Since PtBr_4_ has not brought more substantial benefits for the further copolymerization experiments, it was decided to continue with CuBr_2_/**TPMA** for further experiments although the fact for operation under aerobic conditions could bring some features for other purposes in practice. Furthermore, the experimentally determined number average molecular weight (M_n_) is higher compared to the theoretical number average molecular weight (*M*_n_^th^) as shown in Table 1 for **poly‐1** using short (3 h) and longer irradiation time (24 h), for **poly‐2** only if longer irradiation time (24 h) was selected for NIR‐mediated ATRP, and for **poly‐3** (48 h irradiation). In case of **poly‐2**, the *M*_n_^th^ ‐value is higher compared to the experimentally determined number average molecular weight (M_n_) if a short irradiation time (6 h) was used for the NIR‐mediated ATRP (Table 1, run 2).

**FIGURE 4:**
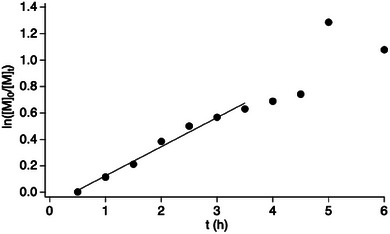
Investigation of 4‐(4‐methacryloyloxyphenyl)butan‐2‐one (**2**) in NIR‐mediated ATRP in a time interval between 30 min and 6 h showing the dependence between monomer consumption (gravimetric determined by isolation of polymer). This enabled to plot data according to first order kinetics as ln([M]_0_/[M]_t_) determined as function of the time (t) yield on isolated **poly‐2** from 2 mL of the irradiated polymerization mixture each.

To get a deeper insight into the high reactivity of **2**, NIR‐mediated ATRP of **2** was investigated as a function of the polymerization time in a time frame up to 6 h. Therefore, **poly‐2** samples were isolated from the polymerization mixture every 30 min up to 6 h exposure time (Figure 4). As expected, yield on **poly‐2** increases during irradiation as shown in Figure 4. One can see a linear dependence of the monomer consumption following first order kinetics for at least 3 h. In this period, formation of polymer occurred quite fast indicating a high reactivity of this monomer in the NIR‐mediated photo‐ATRP protocol. Data deviate after 3 h from first order kinetics, which can be caused by increase of viscosity leaving ideal polymerization conditions. A plot of ln([M]_0_/[M]_t_) as function of time in a frame between 30 min and 24 h makes this clearer (data not shown).

**FIGURE 5:**
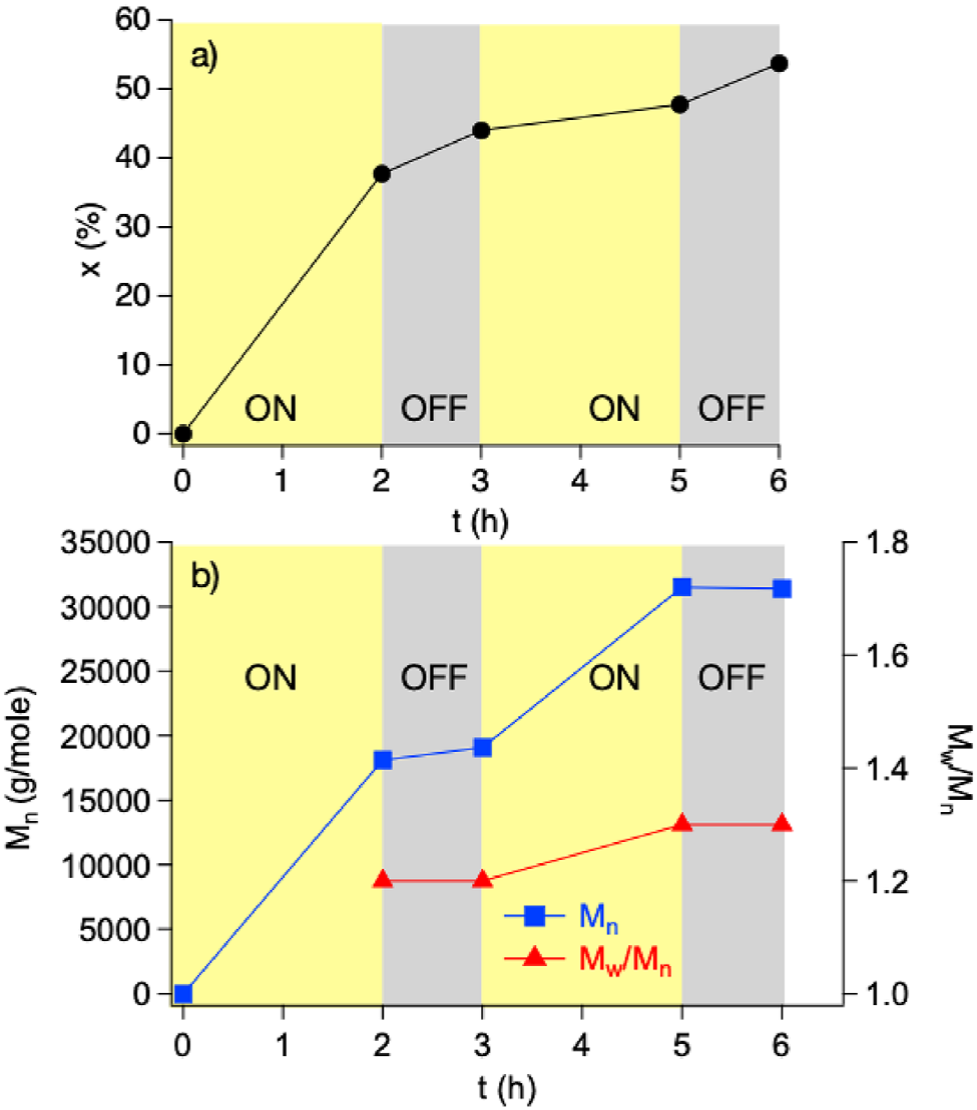
NIR‐mediated ATRP of 4‐(4‐methacryloyloxyphenyl)butan‐2‐one (**2**) during irradiation with light for 2 h followed by a first dark period for 1 h, followed by a second irradiation with NIR‐light for further 2 h, followed by a second dark period for 1 h, a) yield on isolated **poly‐2**, b) number average molecular weight (*M*_n_) and dispersity (*Ð = M_w_/M_n_*) of **poly‐2** isolated after each irradiation period and after each dark period.

GPC investigation of **poly‐2** samples brought more insight about the polymer isolated. The number average molecular weight (*M*_n_) of **poly‐2** continuously increases during irradiation (Figure 5a). Dispersity similarly follows showing values of about 1.2 at exposure time ≳2 h (Figure 5b). These values are slightly higher at shorter irradiation time (30–90 min) corresponding to shorter conversion. A plot of both the experimentally determined number average molecular weight (*M*_n_) and theoretical number average molecular weight (*M*_n_^th^) indicates slightly higher values for experimental *M*_n_ (Figure 5a). This indicates a slight decrease of the initiation efficiency *I*_eff_ with conversion though these changes moderately proceed. However, there does not exist a recognizable dependence at conversions >70% (data not shown), which may be caused by the strong increased viscosity. Data exhibited a strong fluctuation, which may be caused by the change of experimental conditions caused by the higher viscosity—just to mention one possible explanation. This can lead to generation of artifacts by stirring of viscous solutions upon exposure.

Dispersity moderately decreases from 1.3 to 1.2 while it remains there up to a conversion of at least 50% (Figure 5b). Higher conversions connect to an increase of this parameter. This may lead to a decrease of the initiator efficiency.

**FIGURE 6:**
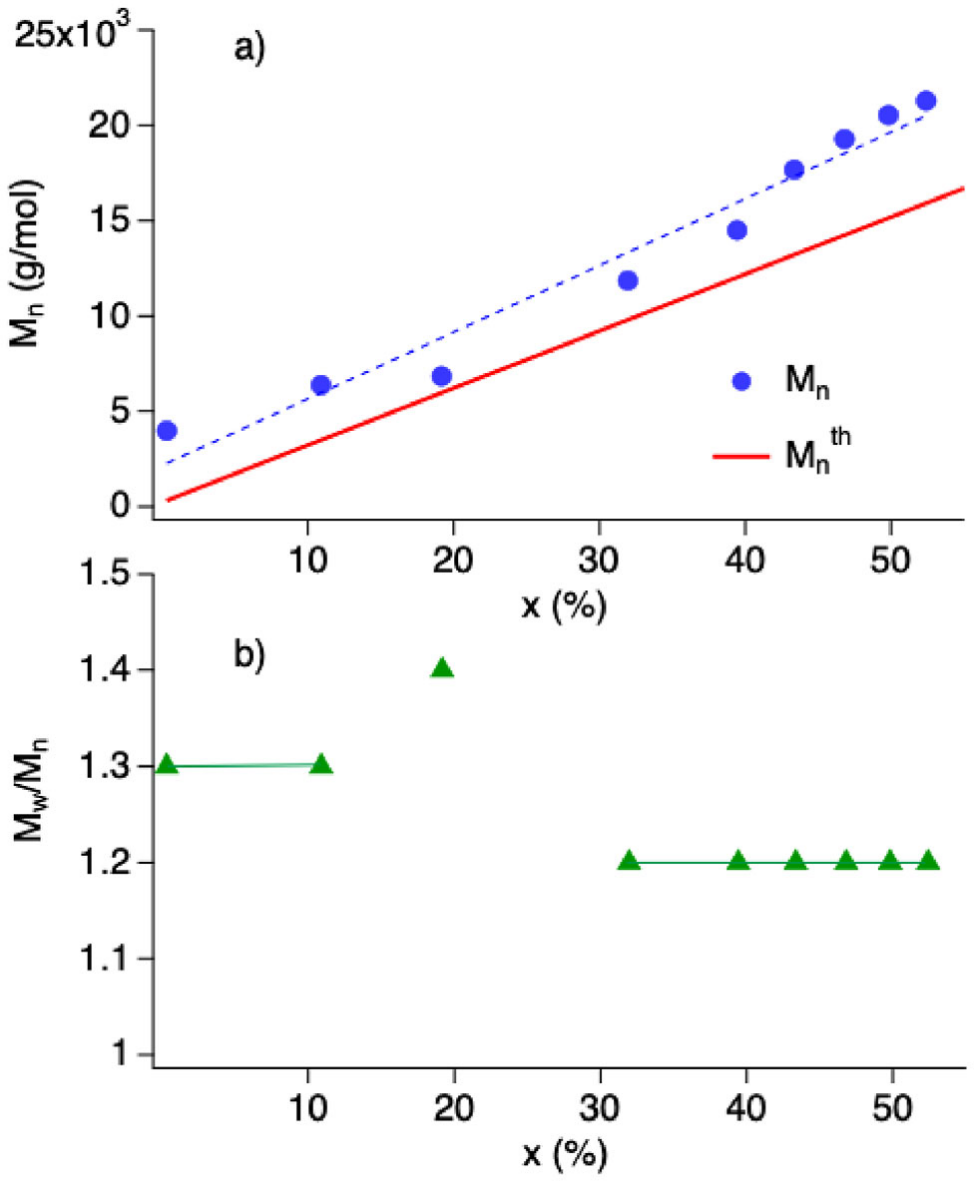
Investigation of 4‐(4‐methacryloyloxyphenyl)butan‐2‐one (**2**) in NIR‐mediated ATRP in a time interval between 30 min and 6 h showing a) the dependence of the experimental (●) and theoretical (**—** solid red line) number average molecular weight (*M*_n_) of **poly‐2** as a function of conversion (x), and b) dependence of dispersity *M*_w_/*M*_n_ of **poly‐2** on conversion.

ON/OFF experiments provide a deeper insight especially regarding a possible polymerization reaction during the dark period. Here, only remaining activator can contribute to polymer formation in an ATRP scheme while previous investigations showed almost back formation of the deactivator using monomer **1** [29]. Figure 6 shows the yield (Figure 6a), number average molecular weight (*M*_n_), and dispersity (*Ð = M_w_/M_n_*) of **poly‐2** (Figure 6b) obtained during NIR‐mediated ATRP where irradiation with NIR‐light for 2 h followed by stirring with no exposure for 1 h. The experiment continued with a further cycle of irradiation and a second dark period. The results depicted in Figure 6a show a significantly higher increase in the yield on **poly‐2** after the first irradiation period compared to the second exposure cycle. Interestingly, *M*_n_ significantly increased during both irradiation periods (Figure 6b). As expected, *M*_n_ remains higher after the second irradiation period compared to the value for *M*_n_ obtained after the first irradiation period (Figure 6b). Polymerization also proceeds in both dark periods as shown by slight increase in yield during the dark periods (Figure 6a). This indicates that deactivation according to Equation 10 and Equation 11, respectively competes with Equation 12 indicating that not all polymer radicals deactivate according to ATRP mechanism. A certain fraction of polymer radicals participates in classical termination (Equation 12), resulting in polymer with higher and lower molecular weight, which is typical for conventional radical polymerization. Furthermore, dispersity only slightly increases from 1.2 to 1.3 during the second irradiation period, although dispersity remains constant during the dark periods (Figure 6b). This confirms results of Figure 5b. The significant increase in the number average molecular weight during both irradiation periods differs from low increase of the number average molecular weight during the dark periods. This clearly shows that light is necessary for formation of **poly‐2**. For comparison, in case of monomer **1**, the conversion on monomer in the dark period was negligible indicating that *k*_d1_/*k*_d2_≈0. The structure of **2**, and therefore, the respective structure of the polymer radical, may mainly cause the increase in the molecular weight during the dark periods.

Moreover, a polymerization time of 6 h was also selected for the NIR‐mediated ATRP homopolymerization of the methyl 9‐hydroxy‐10‐(methacryloyloxy) octadecanoate / methyl 9‐(methacryloyloxy)‐10‐hydroxy octadecanoate isomer mixture (**3a**/**3b**) resulting in only 2% yield on **poly‐3**. Therefore, the polymerization time was increased to 48 h for NIR‐mediated ATRP of the isomer mixture **3a**/**3b** resulting in a higher degree of polymerization (*X*_n_), higher dispersity (*Ð*), and slightly higher yield for **poly‐3** after 48 h irradiation compared to **poly‐1** after 24 h irradiation (Table 1). However, yield on **poly‐3** after 48 h irradiation was significantly lower compared to the yield on **poly‐2** after 24 h irradiation. This shows that the reactivity of the isomer mixture **3a**/**3b** comprising long aliphatic chain is significantly lower compared to the reactivity of the aromatic monomer **2**. It gives a first hint that the structure of copolymers made from **1**, **2**, and **3** may differ as well.

### 2.3 Synthesis of Block Copolymers by NIR‐mediated ATRP

Copolymerization experiments start with **poly‐1** as macroinitiator in combination with either bio‐based monomer **2** comprising a phenyl ring or the bio‐based monomer **3a**/**3b** isomer mixture comprising longer aliphatic chain. Table 2 summarizes the results. The copolymer **poly(1‐*b*‐2)** obtained in 64% yield appeared partially insoluble in THF. Therefore, it is not suitable for GPC investigation. Obviously, the structure of **2** causes this unexpected phenomenon. Furthermore, copolymerization of **poly‐1** with the bio‐based monomer **3a**/**3b** isomer mixture results in the soluble block copolymer **poly(1‐*b*‐3)** in 17% yield. GPC investigation of **poly(1‐*b*‐3)** indicates a chain extension of the **poly‐1** block composed of 125 methyl methacrylate segments by the **poly‐3** block comprising 47 methyl 9‐hydroxy‐10‐(methacryloyloxy) octadecanoate / methyl 9‐(methacryloyloxy)‐10‐hydroxy octadecanoate (**3a**/**3b**) segments (Table 2). This shows that the second polymer block is shorter compared to the first polymer block in case of **poly(1‐*b*‐3)**.

**TABLE 2:** Block copolymers obtained by photo‐ATRP comparing their yield, number‐average molecular weight of the macroinitiator (*M*_n(mac)_), number‐average molecular weight of the block copolymer (*M*_n(b)_), dispersity of macroinitiator (*Đ*_(mac)_), dispersity of the block copolymer (*Đ*_(b)_), polymerization degree of the macroinitiator (*X*_n(mac)_) based on the number‐average molecular weight of the macroinitiator (*M*_n(mac)_), and polymerization degree of the second block in the block copolymer (*X*_n(sb)_) based on the number‐average molecular weight of the block copolymer (*M*_n(b)_). Exposure time was 24 h for most copolymerization experiments except for **poly(3‐*b*‐1)** that operated with 48 h irradiation.

block copolymer	Yield (%)	M_n (mac)_ (g/mol)	*Ð*_(mac)_	M_n (b)_ (g/mol)	*Ð*_(b)_	*X*_n (mac)_	*X*_n (sb)_
**poly(1‐b‐2)**	64	12 551	1.2	^a)^	^a)^	125	^a)^
**poly(1‐b‐3)**	17	12 551	1.2	31 268	1.9	125	47
**poly(2‐b‐1)**	56	47 955	1.8	77 327	2.3	206	293
**poly(2‐b‐3)**	22	42 083	1.6	54 512	1.9	181	31
**poly(3‐b‐1)**	4	90 088	1.4	140 414	1.6	226	503
**poly(3‐b‐2)**	44	90 088	1.4	^a)^	^a)^	226	^a)^

^a)^ partially insoluble in THF.

Moreover, copolymerization of **poly‐2** was carried out with the monomers **1** and **3** resulting in 56% yield on **poly(2‐*b*‐1)** and 22% yield on **poly(2‐*b*‐3)**. GPC investigation of the copolymers shows a chain extension of the poly‐**2** block containing 206 monomer **2** segments by 293 segments of monomer **1** in case of **poly(2‐*b*‐1)**. Furthermore, chain extension of the **poly‐2** block comprising 181 monomer **2** units by the bio‐based monomer **3** resulted in a second block with only 31 monomer units substituted with longer alkyl chain in the block copolymer **poly(2‐*b*‐3)**. This gives a hint to a lower reactivity of **3a**/**3b** compared to both monomers **1** and **2** considering the respective macroinitiator.

Interestingly, chain extension of the **poly‐3** block comprising 226 monomer units of **3** resulted in block copolymerization to **poly(3‐*b*‐1)** with 503 monomer units of **1**. This indicates a higher amount on monomer **1** segments in the second block in case of both block copolymers **poly(2‐*b*‐1)** and **poly(3‐*b*‐1)** that may be attributed to higher mobility of the small monomer **1**. However, the block copolymer **poly(3‐*b*‐2)** is insoluble in THF, and therefore, it is not suitable for GPC measurements as already discussed for the copolymer **poly(1‐*b*‐2)**. It is evident, that the use of **2** in the second block leads to a material that is insoluble in THF, and therefore, these copolymers cannot be treated in a GPC operated with THF.

**FIGURE 7:**
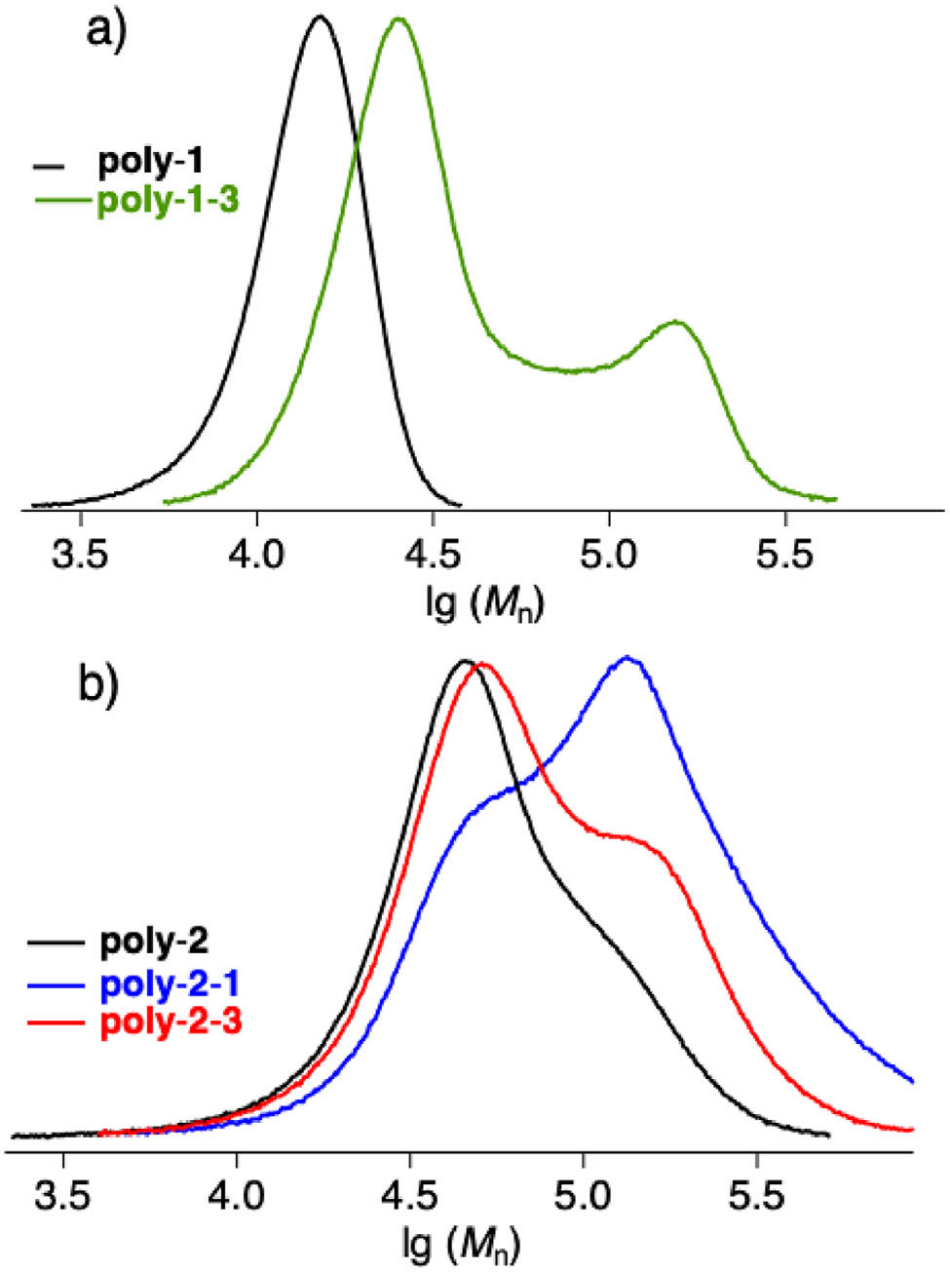
Plot of the relative intensities for the refractive index (RI) with respect to the decadic logarithm of the molecular weight. Data were taken in THF.

Figure 7 plots the relative RI intensities with respect to the decadic logarithm of *M*_n_. The polymer **poly**‐**1** shows a quite unified distribution of *M*_n_, Figure 7a. Block copolymerization with **3** resulted in a bimodal curve where the first peak indicates a shift to higher molecular weight approving controlled polymerization between the macroinitiator **poly**‐**1** and **2**. A detailed view to much higher *M*_n_ images a second peak. This could be assigned to increased intermolecular interactions between these block copolymers leading visually in the GPC data treatment to higher molecular weight fractions. This method requires a well solvated molecule, which does not somehow fit here to interpret the data. It is therefore mostly unlikely that this peak above lg(*M*_n_)>5 assigns to the molecular weight of a well solvated polymer tangle‐a prerequisite for the GPC. Data of **poly**‐**1**‐*b*‐**2** in Table 2 support this proposal, showing an incompatibility in THF.

Plotting the data of the block copolymers comprising **2** supports these findings, Figure 7b. It shows the chain extension of **2** with **1** and **3**, respectively. Though, **poly**‐**2**‐**1** exhibits a smaller fraction with no remarkable increase of molecular weight (see the shoulder appearing at similar *M*_n_). Chain extension of poly‐**1** by **3** resulted in only a small increase of molecular weight while the shoulder appeared at nearly similar high molecular weight compared to **poly**‐**2**‐b‐**1**. These signals may be from our point view of associates of the polymers, which may be caused by a certain inhomogeneity, leading to a somehow misinterpreting scenario.

## 3 Conclusion

The bio‐based methacrylates 4‐(4‐methacryloyloxyphenyl)butan‐2‐one (**2**) and the isomer mixture comprising methyl 9‐hydroxy‐10‐(methacryloyloxy) octadecanoate/methyl 9‐(methacryloyloxy)‐10‐hydroxy octadecanoate (**3a**/**3b**) resulted in polymers with a notable higher yield on **poly‐2** compared to poly(methyl methacrylate) (**poly‐1**) while **poly‐3** gave a lower yield compared to **poly‐1** in the homopolymerization experiments. Yield on the copolymer **poly(1‐*b*‐3)** was lower compared to **poly(1‐*b*‐2)** indicating a lower reactivity of **3** compared to **2** in the copolymerization as found in the homopolymerization experiments as well. The block copolymers made from the bio‐based polymer (**poly‐2** or **poly‐3**) as first block result only in soluble block copolymers if either **1** in case of both bio‐based monomers or **3a**/**3b** in case of **poly‐2** was used as second block. Interestingly, the copolymers made either with **poly‐1** or **poly‐3** as first block and using **2** for formation of the second block are soluble in tetrahydrofuran if they were synthesized in the absence of a sensitizer. The presence of the sensitizer may cause further reactions that result in insolubility of the copolymer in tetrahydrofuran. From this we can conclude that the bio‐based methacrylate isomers **3a**/**3b** are better suitable for the copolymerization as second monomer than the bio‐based methacrylate **2**. Future research may bring some more details about the special functionality of the oxobutyl group containing in the bio‐based monomer **2**.

## 4 Experimental Section

### 4.1 Materials

Copper(II) bromide (Alfa Aesar), platinum tetrabromide (Alfa Aesar), ethyl α‐bromo‐phenyl acetate (Sigma Aldrich), tetrabutyl ammonium bromide (Sigma Aldrich), aluminium oxide basic (Carl Roth), and tris(2‐pyridyl)methyl amine (Sigma Aldrich) were used as received. Dichloromethane, triethylamine, methanol, and tetrahydrofurane from Carl Roth were also used as received. Acros Organics served as supplier for dimethyl formamide.

Methacryloyl chloride from Sigma Aldrich was distilled at normal pressure (bp 96.5°C) prior to use in methacrylate synthesis. The inhibitor contained in commercial methyl methacrylate (from Sigma Aldrich) was removed using a column filled with basic aluminum oxide followed by distillation of the monomer (bp 50°C, 213 mbar) before using the methyl methacrylate in polymerization experiments. **Sens** was available as S 2265 from FEW Chemicals GmbH.

### 4.2 Synthesis of Monomers

Supporting Information discloses synthesis of 4‐(4‐methacryloyloxyphenyl)butan‐2‐one (**2**), and methyl 9‐hydroxy‐10‐(methacryloyloxy) octadecanoate / methyl 9‐(methacryloyloxy)‐10‐hydroxy octadecanoate isomer mixture (**3a**/**3b**).

### 4.3 Polymer Synthesis Following Photo‐ATRP Protocol with NIR Radiation

Supporting Information discloses photo‐ATRP of methyl methacrylate (**1**), 4‐(4‐methacryloyloxyphenyl)butan‐2‐one (**2**), and methyl 9‐hydroxy‐10‐(methacryloyloxy) octadecanoate / methyl 9‐(methacryloyloxy)‐10‐hydroxy octadecanoate isomer mixture (**3a**/**3b**) as well as for synthesis of the block copolymers poly(methyl methacrylate‐*b*‐4‐(4 methacryloyloxyphenyl)butan‐2‐one) (**poly**‐**1**‐***b***‐**2**), poly(4‐(4‐methacryloyloxyphenyl)butan‐2‐one‐b‐methyl methacrylate) (**poly(2‐*b*‐1)**), poly(methyl methacrylate‐*b*‐ (methyl 9‐hydroxy‐10‐(methacryloyloxy) octadecanoate / methyl 9‐(methacryloyloxy)‐10‐hydroxy octadecanoate) (**poly(1‐*b*‐3)**), poly(4‐(4‐methacryloyloxyphenyl)butan‐2‐one)‐*b*‐(methyl 9‐hydroxy‐10‐(methacryloyloxy) octadecanoate / methyl 9‐(methacryloyloxy)‐10‐hydroxy octadecanoate) (**poly(2‐*b*‐3)**), and poly((methyl 9‐hydroxy‐10‐(methacryloyloxy) octadecanoate / methyl 9‐(methacryloyloxy)‐10‐hydroxy octadecanoate)‐*b*‐methyl methacrylate) (**poly(3‐*b*‐1)**), and poly((methyl 9‐hydroxy‐10‐(methacryloyloxy) octadecanoate / methyl 9‐(methacryloyloxy)‐10‐hydroxy octadecanoate‐*b*‐(4‐(4‐methacryloyloxyphenyl)butan‐2‐one)) (**poly(3‐*b*‐2)**).

### 4.4 Methods

#### 4.4.1 NMR Spectroscopy

A Bruker Fourier 300 NMR spectrometer at 300 MHz was used for measuring the NMR spectra of monomers, homopolymers, and block‐copolymers at 298 K. The frequency was 300 MHz for measuring the ^1^H NMR spectra and 75 MHz for the ^13^C NMR spectra. The monomers and polymers were dissolved in CDCl_3_.

#### 4.4.2 FTIR Spectroscopy

FT‐IR spectra of monomers and polymers were measured with a Bruker Vertex 70 equipped with a Platinum ATR inset.

#### 4.4.3 UV–vis Spectroscopy

A UV–vis spectrometer Evolution 220 from Thermo Scientific was applied for measuring the UV–vis spectra of the dissolved samples.

#### 4.4.4 GPC

The molecular weight and molecular weight distribution of the polymers obtained were investigated with a Viscotek GPC‐max from Malvern Instruments that was equipped with a column oven (Malvern, 35°C) containing two columns (T6000M, General Mixed, Org 300×8.0 mm, Viscotek). A refractive index detector VE 3580 RI Detector from Viscotek was used for detection of the polymer. Tetrahydrofuran (THF) was used as eluent with a flow rate of 1 mL·min^−1^. Poly(methyl methacrylate) standards (PMMA: 1850, 6830, 20 100, 73 200, 218 000, 608 000, and 1 050 000 g·mol^−1^) from Shodex were applied for calibration. Dried polymer samples (10 mg) were dissolved in THF (2 mL) for 24 h, and the polymer solutions were filtered through PTFE syringe filters (0.2 µm) before injection of 50 µL polymer solution. Each polymer sample was injected twice. The molecular weight was analyzed relative to the PMMA standards called above.

#### 4.4.5 DSC

A Differential Scanning Calorimeter DSC Q 2000 from TA Instruments equipped with a liquid nitrogen cooling system was used to investigate the melting behavior of the liquid monomers and to determine the glass transition temperature of the polymers obtained. Indium (164.5°C) and zinc (419.7°C) were used for calibration of the DSC. For the DSC measurements, each sample was placed in an aluminum T^zero^ pan, which was hermetically sealed with a T^zero^ lid. The measurements were carried out in a temperature range between ‐70°C and 100°C using cooling and heating rates of 5 K/min (monomers) and 10 K/min (polymers), respectively.

## Funding

The authors gratefully acknowledge the ZIM program of the AIF Germany grant number KK5297502BU1 (measurements on the polymers) as well as the European Union, the MWIDE NRW Germany, the Ministerie van Economische Zaken en Klimaat Netherlands, the provinces of Limburg, Gelderland, Noord‐Brabant und Overijssel that co‐financed the INTERREG‐Program Deutschland‐Nederland of the EU (grant project DNL‐HIT) (polymer synthesis).

## Conflicts of Interest

The authors declare no conflict of interest.

## Supporting information




**Supporting File**: marc70120‐sup‐0001‐SuppMat.docx

## Data Availability

The data that support the findings of this study are available from the corresponding Author Veronika Strehmel upon reasonable request.
