# Supplementary material for: Synthesis of Polymers From Bio‐Based Methacrylates Comprising Aromatic or Aliphatic Structures Using NIR‐Mediated ATRP
Source: Macromol Rapid Commun. 2025 Nov 9;47(1):e00718. doi: 10.1002/marc.202500718 (PMC12784184; doi:10.1002/marc.202500718)
Supplement: Supplementary file 1 — Supporting File: marc70120‐sup‐0001‐SuppMat.docx [file MARC-47-e00718-s001.docx]

Supporting Information

Synthesis of Polymers from Bio-based Methacrylates Comprising Aromatic or Aliphatic Structures Using NIR-Mediated ATRP

Nicolai Meckbach, Lea Viktoria Rubbert, Bernd Strehmel*, and Veronika Strehmel*

*corresponding authors

**Experimental**

S1. Synthesis of the Monomers

S1.1. Synthesis of 4-(4-Methacryloyloxyphenyl)butan-2-one (**2**)

The 4-(4-methacryloyloxyphenyl)butan-2-one was synthesized from 4-(4-hydroxyphenyl)

butan-2-one and methacryloyl chloride in dichloromethane in the presence of triethylamine as described in the literature.^[ S1, S2]^ The 4-(4-hydroxyphenyl)butan-2-one (82.1 g) was dissolved in dichloromethane (300 ml), and the solution was cooled to -3°C before freshly distilled methacryloyl chloride (58.6 ml) dissolved in 50 ml dichloromethane was slowly dropped during stirring into the solution under argon within 3 hours resulting in an increase of the reaction mixture temperature to 8 °C. After addition of the methacryloyl chloride was completed, the reaction mixture was further stirred for 16 h. Filtration of the yellow suspension followed by washing the filter cake with 50 ml dichloromethane, unification of the dichloromethane solutions, washing the unified dichloromethane solution with 100 ml hydrochloric acid each three times at first, with potassium carbonate solution (1 M, 100 ml each) two times second, and with 100 ml water each three times third. Then, the solvent was evaporated at 25 °C during reducing the pressure from 350 mbar to 10 mbar. This resulted in the 4-(4-methacryloyloxyphenyl)butan-2-one raw product (103.3 g) in 90.3 % yield. The raw product was purified by column chromatography using ethyl acetate as mobile phase and basic aluminium oxide as stationary phase resulting in 4-(4-methacryloyloxyphenyl)butan-2-one as clear slightly amber-like colored liquid (97.65 g) in 84.1 % yield. Then, the 4-(4-methacryloyloxyphenyl)butan-2-one was dissolved in a *n*-hexane ethyl acetate (4:1 = v:v) mixture, and the resulting slightly turbid solution was placed in a refrigerator at -20°C. The crystals formed were filtrated followed by drying at 22 °C under vacuum (0.08 mbar) resulting in 45.1 % yield on purified 4-(4-methacryloyloxyphenyl)butan-2-one (**2**, 52.34 g, m.p. 29 °C).^[ S1, S2]^

S1.2. Synthesis of Methyl 9-Hydroxy-10-(Methacryloyloxy) Octadecanoate / Methyl 9-(Methacryloyloxy)-10-Hydroxy Octadecanoate Isomer Mixture (**3a**/**3b**)

The methyl 9-hydroxy-10-(methacryloyloxy) octadecanoate / methyl 9-(methacryloyloxy)-10-hydroxy octadecanoate isomer mixture (**3a**/**3b**) was synthesized in a three-step reaction starting with esterification of oleic acid with methanol, followed by epoxidation of the methyl oleate in the second step, and epoxy ring opening reaction of the 9,10-epoxy methyl octadecanoate using methacrylic acid in the third step as described in the literature.^[61-S3]^

Methyl Oleate: Oleic acid was purified by deep temperature crystallization of commercial oleic acid (140 g) dissolved in acetone (700 ml) at -30 °C. After cooling the mixture at -30 °C for 8 h, the crystallized saturated fatty acids (44 g) were separated from the solution followed by evaporation of the solvent resulting in 96 g oleic acid containing higher unsaturated fatty acids. This oleic acid fraction was dissolved in acetone (615 ml) again, and the resulting solution was cooled at -30 °C again for 24h resulting in crystallization of the oleic acid. The oleic acid crystals were separated from the solution followed by removing of remaining acetone after melting the crystals at 40 °C and 350 mbar and drying the resulting liquid oleic acid under reduced pressure (0.69 mbar). This resulted in purified oleic acid in 41 % yield that was used for the synthesis of methyl oleate.

Methyl oleate was synthesized from 40 g purified oleic acid and 21 ml methanol in the presence of 1.5 ml of concentrated sulfuric acid during stirring at 70 °C for 24 h. Then, the methanol excess was removed by distillation resulting in a two-phase mixture. Furthermore, a saturated calcium chloride solution (55 g calcium chloride dissolved in 750 ml water) was added to this mixture at 40 °C and stirred at 40 °C for 1h followed by separation of the organic phase and the aqueous phase. The aqueous phase was extracted with diethyl ether. The organic phases were unified followed by drying over magnesium sulfate. The resulting clear organic solution was filtrated, and the diethyl ether was evaporated at 40 °C and 830 mbar using a rotational evaporator followed by drying the product at this temperature during reducing the pressure to 0.72 mbar. This resulted in methyl oleate (34 g) in 81 % yield.

Methyl 9,10-Epoxy Octadecanoate: Methyl oleate (30 ml) was dissolved in formic acid (8.4 ml), and the solution was cooled to 0 °C during stirring under argon followed by slowly dropping of hydrogen peroxide (% 50, 12.9 ml) into the stirred cooled mixture. After addition of hydrogen peroxide was completed, the reaction mixture was continuously stirred under argon at 0 °C for an additional hour. Then, the reaction mixture was allowed to slowly warm up to room temperature during stirring followed by continuously stirring at room temperature for further 5 h. After the reaction was finished, 300 ml water were added to the reaction mixture followed by slowly addition of aqueous sodium hydrogen carbonate solution (19.2 g sodium hydrogen carbonate dissolved in 200 ml water) to the reaction mixture during stirring. After foam formation was over, the organic phase was separated from the aqueous phase. The aqueous phase was washed with 300 ml ethyl acetate. The organic phases were unified, and the unified organic phases were washed with water (600 ml). Then, the organic phase was dried over magnesium sulfate resulting in a clear organic solution. After filtration of the magnesium sulfate, the solvent was evaporated at 30 °C and 155 mbar at first, and the residue was dried at 30 °C and 0.64 mbar resulting in methyl 9,10-epoxy octadecanoate (24.2 g) in 87,6 % yield.

Methyl 9-Hydroxy-10-(Methacryloyloxy) Octadecanoate / Methyl 9-(Methacryloyloxy)-10-Hydroxy Octadecanoate Isomer Mixture (**3a**/**3b**): The synthesis of the methyl 9-hydroxy-10-(methacryloyloxy) octadecanoate / methyl 9-(methacryloyloxy)-10-hydroxy octadecanoate isomer mixture (**3a**/**3b**) was carried out as described in the literature.^[ S3]^ Hydroquinone (20 mg) and diazabicyclooctane (DABCO, 20 mg) were added to 9,10-epoxy octadecanoate (10 g) during stirring followed by slowly dropping of methacrylic acid (4.1 ml, stabilized with methyl hydroquinone) under stirring into the mixture. Then, the resulting mixture was heated to 95 °C during continuously stirring, and the mixture was stirred at this temperature for further 24 h. After cooling the reaction mixture down to room temperature, 100 ml water were added. Then, the organic phase was separated from the aqueous phase. The aqueous phase was extracted with 100 ml diethyl ether. The organic phases were unified followed by drying the unified organic phase over magnesium sulfate. After separation of the magnesium sulfate by filtration, the drying agent was washed with diethyl ether. The organic phases were unified, and the solvent was evaporated at 33 °C and 800 mbar resulting in the methyl 9-hydroxy-10-(methacryloyloxy) octadecanoate / methyl 9-(methacryloyloxy)-10-hydroxy octadecanoate (**3a**/**3b**) raw product. The raw product was dissolved in 20 ml ethyl acetate for column chromatographic purification using a column filled with aluminium oxide (6 g aluminium oxide relative to 1 g raw product) and covered with sea sand. Ethyl acetate (300 ml) was used as eluent for the column chromatographic purification resulting in purified methyl 9-hydroxy-10-(methacryloyloxy) octadecanoate / methyl 9-(methacryloyloxy)-10-hydroxy octadecanoate isomer mixture **3a**/**3b** (10.4 g) in 81,5 % yield.

S2. Polymer Synthesis following photo-ATRP protocol

Photo-ATRP was carried out as described in the literature.^[S4]^ The photo-reactor was equipped with four NIR-LED’s (emission wavelength at 790 nm, light intensity of 100 mW/cm^2^) from Roithner Lasertechnik mounted in an angle of 90° to each other.

The Schlenk flask (25 mL) equipped with a stirrer was evacuated at room temperature and 0.045 mbar for 10 min followed by purging with nitrogen. This procedure was repeated three times before the components used for the photo-ATRP experiments were added to the Schlenk flask as discussed below. After addition of the monomer and photoinitiator system containing solution to the Schlenk flask, it was degassed and purged with nitrogen. Then, the Schlenk tube containing the monomer initiator solution was placed in the center of the photoreactor for irradiation at 790 nm.

S2.1. Photo-ATRP of Methyl Methacrylate (1) using NIR radiation

A Schlenk tube equipped with a stirrer was evacuated at room temperature and 0.045 mbar for 10 min followed by purging with nitrogen. This procedure was repeated three times. Then, the sensitizer S 2265 from FEW Chemicals GmbH (32.82 mg) dissolved in 4.8 ml dimethyl formamide was added to the photoreactor during purging with nitrogen followed by addition of 4.8 ml methyl methacrylate (**1**), 75 µl of a 270 mM tris(2-pyridyl methyl amine) (**TPMA**) solution in dimethyl formamide, 25 µl of a 180 mM CuBr_2_ solution in dimethyl formamide, and 26.25 µl ethyl-a-bromophenyl acetate (**EBPA**). After addition of all components during purging with nitrogen was finished, the Schlenk tube was closed, and irradiation of 9.7 ml reaction volume was started in the photoreactor. After 24 h irradiation, the polymer was precipitated using 10 times of methanol relative to the volume of the polymerization mixture. The precipitated poly(methyl methacrylate) (**poly-1**) was isolated, washed with methanol, and dried. For purification, **poly-1** was dissolved in tetrahydrofuran followed by precipitation into cold methanol, isolation of **poly-1**, and drying under ambient conditions at first and then in a vacuum drying oven at 45 °C resulting in 31.19 % yield on **poly-1** (1.41 g). The ^1^H and ^13^C NMR spectra of **poly-1** are given in Figure S1.

S2.2. Photo-ATRP of 4-(4-Methacryloyloxyphenyl)butan-2-one (**2**) using NIR radiation

A Schlenk tube equipped with a stirrer was evacuated at room temperature and 0.045 mbar for 10 min followed by purging with nitrogen. This procedure was repeated three times. Then, the sensitizer S 2265 (32.82 mg) dissolved in 2 ml dimethyl formamide and **2** (10.45 g) dissolved in 2.8 ml dimethyl formamide were added to the photoreactor during purging with nitrogen followed by addition of 75 µl of a 270 mM **TPMA** solution in dimethyl formamide, 25 µl of a 180 mM CuBr_2_ solution in dimethyl formamide, and 26.25 µl **EBPA**. After addition of all components during purging with nitrogen was finished, the Schlenk tube was closed. Then, the solution (14.9 ml) was irradiated in the photoreactor for 24 h resulting in a high viscous solution. The polymer was precipitated from the viscous solution using 10 times of methanol relative to the volume of the polymerization mixture. The precipitated poly(4-(4-methacryloyloxyphenyl)butan-2-one) (**poly-2**) was isolated and dried. For purification, **poly-2** was dissolved in tetrahydrofuran followed by precipitation into cold methanol. The isolated **poly-2** was dried under ambient conditions at first and then in a vacuum drying oven at 45 °C. This resulted in 8.8 g **poly-2** corresponding to 84.09 % yield on **poly-2**. The ^1^H and ^13^C NMR spectra of **poly-2** are depicted in Figure S2.

For kinetic investigation of this polymerization, the sensitizer S 2265 (32.82 mg) was dissolved in 3.6 ml of dimethyl formamide and **2** (10.45 g) was dissolved in 6 ml of dimethyl formamide to obtain a reaction volume of 19.7 ml that was sufficient to take samples of 1.5 ml each of the polymerizing solution in a time interval of 45 min during 6 h irradiation. The working up procedure was the same as described above.

For ON-OFF experiments, the sensitizer S 2265 (32.82 mg) was dissolved in 3.6 ml of dimethyl formamide, and monomer **2** (10.45 g) was dissolved in 6 ml of dimethyl formamide to obtain a reaction volume of 19.7 ml that was the same as for the kinetic investigation. The resulting solution was put into the Schlenk tube in the photoreactor during purging with nitrogen followed by addition of 75 µl of a 270 mM **TPMA** solution in dimethyl formamide, 25 µl of a 180 mM CuBr_2_ solution in dimethyl formamide, and 26.25 µl **EBPA** during continuously purging the solution with nitrogen. After addition of all components during purging with nitrogen, the Schlenk tube was closed, and the irradiation was started in the photoreactor. After 2 h irradiation with light during stirring, the first sample (1.5 mL) was taken followed by switching off the light for 1 h. The polymer containing in the first sample was immediately precipitated into cold methanol, isolated, washed with cold methanol, and dried. After the polymerizing mixture was 1 h in the dark, the second sample was taken followed by switching on the light again for further 2h. This resulted in proceeding of the polymerization during irradiation. After 2 h irradiation with light, a third sample was taken followed by switching off the light for 1 h again. The polymer was precipitated immediately into cold methanol after taking the sample, isolated, and purified as described above. At the end of the dark period, the polymer was precipitated into cold methanol, isolated, and purified as described above resulting in a fourth polymer sample. Finally, all four polymer samples were dried followed by dissolution in tetrahydrofuran and precipitation into cold methanol. The isolated polymer samples were dried under ambient conditions at first, and finally, in a vacuum drying oven at 45 °C.

S2.3. Photo-ATRP of Methyl 9-Hydroxy-10-(Methacryloyloxy) Octadecanoate / Methyl 9-(Methacryloyloxy)-10-Hydroxy Octadecanoate Isomer Mixture (**3a**/**3b**) Using NIR Radiation

The Schlenk tube equipped with a stirrer was evacuated at room temperature and 0.045 mbar for 10 min followed by purging with nitrogen. This procedure was repeated three times. Then, solutions of the sensitizer S 2265 (8.22 mg) dissolved in 0.4 ml dimethylformamide, 4.48 g (11.2 mmol) of the **3a**/**3b** isomer mixture dissolved in 0.8 ml dimethylformamide, 18.75 µl **TPMA** solution (0.005 mmol **TPMA**), 6.25 µl CuBr_2_ solution (0.001 mmol CuBr_2_), and 6.5 µl (0.03 mmol) **EBPA** were added to the pretreated Schlenk tube in the photoreactor during continuously purging with nitrogen. This resulted in a reaction mixture volume of 5.7 ml for irradiation in the photoreactor. After closing the Schlenk tube, irradiation was carried out for 6 h and 48 h, respectively. After finishing the irradiation, the reaction mixture was dropped into cold methanol (10 times of the volume relative to the reaction mixture). The polymer (**poly-3**) was obtained as highly viscous liquid. **Poly-3** was isolated, washed with further methanol, and dried under vacuum. Further purification was carried out after dissolution of **poly-3** in acetone followed by precipitation into methanol resulting in a high viscous liquid that was isolated and dried under vacuum resulting in 2.45 % yield on **poly-3** (0.11 g) after 6 h irradiation, 10.27 % yield on **poly-3** after 20 h irradiation, and 37.95 % yield on **poly-3** (1.7 g) after 48 h irradiation, respectively. The ^1^H and ^13^C NMR spectra of **poly-3** are shown Figure S3.

S2.4. Synthesis of Poly(methyl methacrylate-*b*-4-(4 methacryloyloxyphenyl)butan-2-one) (**poly-1-*b*-2**)

For the synthesis of the copolymer **poly(1-*b*-2)** in the presence of the sensitizer S2265, **poly-1** (0.3 g) made by photo-ATRP was dissolved in 0.6 mL dimethyl formamide and monomer **2** (1.33 g) dissolved in 0.6 mL dimethyl formamide was added to the Schlenk tube that was placed in the photoreactor and purged with nitrogen. Then, 19.97 µl of a 270 mM **TPMA** solution in dimethyl formamide, 3.3 µl of a 180 mM CuBr_2_ solution in dimethyl formamide, and 4.69 mg of the sensitizer S2265 were added during continuously purging the monomer containing solution with nitrogen. The entire reaction volume of 2.4 mL was irradiated in the photoreactor for 24 h. After finishing the irradiation, a high viscous solution was obtained. The co-polymer was precipitated into cold methanol, isolated, purified as described above, and finally dried in a vacuum drying oven at 45 °C. This resulted in 1.05 g poly(methyl methacrylate-*b*-4-(4-methacryloyloxyphenyl)butan-2-one) (**poly(1-*b*-2)**) corresponding to 64.42 % yield. The NMR spectra of **poly(1-*b*-2)** synthesized in the presence of the sensitizer S2265 are shown in Figure S4. However, as this polymer sample **poly(1-*b*-2)** was not completely soluble in tetrahydrofuran, molecular weight determination using GPC was not possible for this sample of **poly(1-*b*-2)**.

S2.5. Synthesis of Poly(4-(4-Methacryloyloxyphenyl)butan-2-one-*b*-Methyl Methacrylate) (**poly(2-*b*-1)**)

For the synthesis of **poly(2-*b*-1)**, **poly-2** (2.15 g) made by photo-ATRP was dissolved in 3 mL dimethylformamide and methyl methacrylate (**1**) (1.48 g) was added to this solution. The resulting mixture was placed in the photoreactor and purged with nitrogen. Then, 6.57 mg of the sensitizer S2265, 45 µl of a 270 mM **TPMA** solution in dimethyl formamide, and 7.5 µl of a 180 mM CuBr_2_ solution in dimethylformamide were added to this solution as well during continuously stirring and purging with nitrogen. The reaction volume (4.5 mL) was irradiated in the photoreactor for 24 h. After finishing the irradiation, a high viscous solution was obtained. The co-polymer was precipitated into cold methanol, isolated, purified as described above, and finally, dried in a vacuum drying oven at 45 °C. This resulted in 26 % yield on **poly(2-*b*-1)**. The NMR spectra of **poly(2-*b*-1)** are shown in Figure S5. Furthermore, the poly(4-(4 methacryloyloxyphenyl)butan-2-one-*b*- methyl methacrylate) (**poly(2-*b*-1)**) obtained was soluble in THF. GPC investigation resulted in a number average molecular weight of 77327 g/mol and dispersity of 2.3 that is higher compared to **poly-2** used as first block (M_n_ = 47955 g/mol, dispersity = 1.8) in this copolymerization experiment.

S2.6. Synthesis of Poly(methyl methacrylate-*b*- (methyl 9-hydroxy-10-(methacryloyloxy) octadecanoate / methyl 9-(methacryloyloxy)-10-hydroxy octadecanoate) (**poly(1-*b*-3)**)

The Schlenk tube equipped with a stirrer was evacuated at room temperature and 0.045 mbar for 10 min followed by purging with nitrogen. This procedure was repeated three times. Then, **poly-1** (0.76 g) dissolved in 1 ml dimethylformamide, 6.57 mg (0.009mmol) sensitizer S2265 dissolved in 0.5 ml dimethylformamide, 5.38 g (13.5 mmol) monomer **3** isomer mixture, 45 µl **TPMA** solution (0.012 mmol **TPMA**) in dimethylformamide, and 7.5 µl CuBr_2_ (0.0013 mmol CuBr_2_) solution in dimethylformamide were added during continuously purging with nitrogen. After closing the Schlenk tube, irradiation of the entire reaction volume (6.9 ml) was carried out for 24 h. After finishing the irradiation, the entire reaction mixture was dropped into cold methanol covering a volume that corresponded to 10 times of the reaction mixture volume. The block copolymer obtained was isolated, washed with cold methanol, and dried resulting in 1.05 g of poly(methyl methacrylate-*b*-(methyl 9-hydroxy-10-(methacryloyloxy) octadecanoate / methyl 9-(methacryloyloxy)-10-hydroxy octadecanoate) (**poly(1-*b*-3)**) corresponding to 17.1 % yield. Further purification of **poly(1-*b*-3)** was carried out by dissolution in tetrahydrofuran followed by precipitation into cold methanol, isolation of the polymer, washing the polymer with further cold methanol, and drying the polymer at 45 °C and 0.045 mbar. The ^1^H NMR spectrum of the block copolymer poly(methyl methacrylate-*b*-(methyl 9-hydroxy-10-(methacryloyloxy) octadecanoate / methyl 9-(methacryloyloxy)-10-hydroxy octadecanoate) (**poly(1-*b*-3)**) is depicted in Figure S6.

S2.7. Synthesis of Poly(4-(4-methacryloyloxyphenyl)butan-2-one)-*b*-(methyl 9-hydroxy-10-(methacryloyloxy) octadecanoate / methyl 9-(methacryloyloxy)-10-hydroxy octadecanoate) (**poly(2-*b*-3)**)

The Schlenk tube equipped with a stirrer was evacuated at room temperature and 0.045 mbar for 10 min followed by purging with nitrogen. This procedure was repeated three times. Then, **poly-2** (1.89 g; 0.045 mmol) dissolved in 2.5 ml dimethylformamide, 6.57 mg (0.009mmol) sensitizer S2265 dissolved in 0.5 ml dimethylformamide, 5.38 g (13.5 mmol) methyl 9-hydroxy-10-(methacryloyloxy) octadecanoate / methyl 9-(methacryloyloxy)-10-hydroxy octadecanoate isomer mixture, 45 µl **TPMA** solution (0.012 mmol **TPMA**) in dimethylformamide and 7.5 µl CuBr_2_ (0.0013 mmol CuBr_2_) solution in dimethylformamide were added during continuously purging with nitrogen. After closing the Schlenk tube, irradiation of the entire reaction volume (8.4 ml) was carried out for 24 h. After finishing the irradiation, the entire reaction mixture was dropped into cold methanol covering a volume that corresponded to 10 times of the reaction mixture volume. The block copolymer obtained was isolated, washed with cold methanol, and dried resulting in 22.28 % yield on **poly(2-*b*-3)** (1.62 g). Further purification of the polymer was carried out by dissolution in tetrahydrofuran followed by precipitation into cold methanol, isolation of the polymer, washing the polymer with further cold methanol, and drying the polymer at 45 °C and 0.045 mbar. The NMR spectra of the block copolymer poly(4-(4-methacryloyloxyphenyl)butan-2-one)-*b*-(methyl 9-hydroxy-10-(methacryloyloxy) octadecanoate / methyl 9-(methacryloyloxy)-10-hydroxy octadecanoate) (**poly(2-*b*-3)**) are depicted in Figure S7.

S2.8. Synthesis of Poly( (methyl 9-hydroxy-10-(methacryloyloxy) octadecanoate / methyl 9-(methacryloyloxy)-10-hydroxy octadecanoate)-*b*-methyl methacrylate) (**poly(3-*b*-1)**)

The Schlenk tube equipped with a stirrer was evacuated at room temperature and 0.045 mbar for 10 min followed by purging with nitrogen. This procedure was repeated three times. Then, poly(*methyl 9-hydroxy-10-(methacryloyloxy) octadecanoate / methyl 9-(methacryloyloxy)-10-hydroxy octadecanoate)* (0.06 g) dissolved in 0.8 ml dimethylformamide, 0.52 mg (0.0007mmol) sensitizer S2265 dissolved in 0.2 ml dimethylformamide, 2 g methylmethacrylate, 2.8 µl **TPMA** solution (0.0007 mmol **TPMA**) in dimethylformamide, and 0.4 µl CuBr_2_ (0.00007 mmol CuBr_2_) solution in dimethylformamide were added during continuously purging with nitrogen. After closing the Schlenk tube, irradiation of the reaction volume (3.1 ml) was carried out for 48 h. After finishing the irradiation, the co-polymer was precipitated into cold methanol, isolated, purified as described above, and finally dried in a vacuum drying oven at 45 °C. This resulted in 3.64 % yield on **poly(3-*b*-1)** (75 mg). Further purification was carried out by dissolution in tetrahydrofuran followed by precipitation into cold methanol, isolation of the polymer, washing the polymer with further cold methanol, and drying the polymer at 45 °C and 0.045 mbar. The ^1^H and ^13^C NMR spectra of the block copolymer poly((methyl 9-hydroxy-10-(methacryloyloxy) octadecanoate / methyl 9-(methacryloyloxy)-10-hydroxy octadecanoate)-*b*-methyl methacrylate) **poly(3-*b*-1)** are depicted in Figure S8.

S2.9. Synthesis of Poly( (methyl 9-hydroxy-10-(methacryloyloxy) octadecanoate / methyl 9-(methacryloyloxy)-10-hydroxy octadecanoate-*b*-(4-(4-methacryloyloxyphenyl)butan-2-one)) (**poly(3-*b*-2)**)

The Schlenk tube equipped with a stirrer was evacuated at room temperature and 0.045 mbar for 10 min followed by purging with nitrogen. This procedure was repeated three times. Then, poly(*methyl 9-hydroxy-10-(methacryloyloxy) octadecanoate / methyl 9-(methacryloyloxy)-10-hydroxy octadecanoate)* (0.06 g) dissolved in 0.8 ml dimethylformamide, 0.52 mg (0.0007mmol) sensitizer S2265 dissolved in 0.2 ml dimethylformamide, 4.6 g (20 mmol) 4-(4-methacryloyloxyphenyl)butan-2-one (**2**), 2.8 µl **TPMA** solution (0.0007 mmol **TPMA**) in dimethylformamide, and 0.4 µl CuBr_2_ (0.00007 mmol CuBr_2_) solution in dimethylformamide were added during continuously purging with nitrogen. After closing the Schlenk tube, irradiation of the reaction volume (5.7 ml) was carried out for 24 h. After finishing the irradiation, the gel-like co-polymer was stirred with cold methanol, isolated, purified as described above, and finally dried in a vacuum drying oven at 45 °C. This resulted in 44.21 % yield on **poly(3*-b*-2)** (2.06 g). Only a small amount of **poly(3-*b*-2)** dissolved in tetrahydrofuran. Precipitation of the polymer fraction dissolved in tetrahydrofuran into cold methanol, isolation, and drying the precipitate resulted in 33.57 mg of the tetrahydrofuran soluble **poly(3-*b*-2)** fraction. The **poly(3-*b*-2)** fraction, which was insoluble in tetrahydrofuran, was stirred with methanol for 12 h, isolated, and dried at 45 °C and 0.045 mbar. This shows that the copolymer **poly(3-*b*-2)** is composed of 1.63 % tetrahydrofuran soluble fraction and 98.37 % insoluble fraction. The ^1^H NMR spectrum of the tetrahydrofuran soluble fraction of **poly(3-*b*-2)** is depicted in Figure S9.

S3. NMR Spectra

a)

b)

**Figure S1.** NMR spectra of poly(methyl methacrylate) (**poly-1**) dissolved in CDCl_3_,

a) ^1^H NMR spectrum; b) ^13^C NMR spectrum.

a)

b)

**Figure S2.** NMR spectra of poly(4-(4-methacryloyloxyphenyl)butan-2-one) (**poly-2**) dissolved in CDCl_3_, a) ^1^H NMR spectrum; b) ^13^C NMR spectrum.

a)

b)

**Figure S3.** NMR spectra of **poly-3** dissolved in CDCl_3_, a) ^1^H NMR spectrum; b) ^13^C NMR spectrum.

**Figure S4.** NMR spectra of **poly(1-*b*-2)** dissolved in CDCl_3_, a) ^1^H NMR spectrum; b) ^13^C NMR spectrum.

**Figure S5.** NMR spectra of **poly(2-*b*-1)** dissolved in CDCl_3_, a) ^1^H NMR spectrum; b) ^13^C NMR spectrum.

**Figure S6.** ^1^H NMR spectrum of **poly(1-*b*-3)** dissolved in CDCl_3_.

**Figure S7.** NMR spectra of **poly(2-*b*-3)** dissolved in CDCl_3_, a) ^1^H NMR spectrum; b) ^13^C NMR spectrum.

**Figure S8.** NMR spectra of **poly(3-*b*-1)** dissolved in CDCl_3_, a) ^1^H NMR spectrum; b) ^13^C NMR spectrum.

**Figure S9.** NMR spectrum of the tetrahydrofuran soluble fraction of **poly(3-*b*-2)** dissolved in CDCl_3_.

S4. References

[S1] M. Heinz, M. Kepkow, B. Strehmel, V. Strehmel, "Synthesis and Photo-Initiated Radical Polymerization of Methacrylates Derived from Bio-Based ω-Hydroxycarboxylic Acids," *Appl. Res.* 3 (2024): e202300124. <https://doi.org/10.1002/appl.202300124>

[S2] T. G. Ribelli, F. Lorandi, M. Fantin, K. Matyjaszewski, "Atom Transfer Radical Polymerization: Billion Times More Active Catalysts and New Initiation Systems," *Macromol. Rapid Commun.* 40 (2019): 1800616. <https://doi.org/10.1002/marc.201800616>

[S3] M. Heinz, M. Kepkow, V. Strehmel, "Statistical Copolymers Comprising Bio-Based Aromatic Methacrylate Segments and Their Influence on the Glass Transition Temperature," *Macromol. Chem. Phys.* 224 (2023): 2300031. <https://doi.org/10.1002/macp.202300031>

[S4] C. Kütahya, C. Schmitz, V. Strehmel, Y.Yagci, B. Strehmel, "Near-Infrared Sensitized Photoinduced Atom-Transfer Radical Polymerization (ATRP) with a Copper(II) Catalyst Concentration in the ppm Range," *Angew. Chem., Int. Ed.* 57 (2018): 7898-7902. <https://doi.org/10.1002/anie.201802964>
